# Low platelet count at diagnosis of anti-neutrophil cytoplasmic antibody-associated vasculitis is correlated with the severity of disease and renal prognosis

**DOI:** 10.1007/s10238-024-01333-z

**Published:** 2024-04-05

**Authors:** Yanli Jin, Fangyuan Wang, Jiale Tang, Liying Luo, Lingyu Huang, Fangyu Zhou, Enyu Qi, Xinyue Hu, Shuanglinzi Deng, Huan Ge, Yuanyuan Jiang, Juntao Feng, Xiaozhao Li

**Affiliations:** 1 Department of Nephrology, Xiangya Hospital, Central South University, No.87 Xiangya Road, Kaifu District, Changsha, Hunan China; 2 Center of Respiratory Medicine, Xiangya Hospital, Central South University, Changsha, China; 3 Department of Laboratory Medicine, Xiangya Hospital, Central South University, Changsha, China

**Keywords:** Platelet count, Antineutrophil cytoplasmic antibody (ANCA)-associated vasculitis, End-stage renal disease, Survival, Prognosis

## Abstract

Antineutrophil cytoplasmic antibody-associated vasculitis (AAV) is an autoimmune disease that involves inflammation of blood vessels. There is increasing evidence that platelets play a crucial role not only in hemostasis but also in inflammation and innate immunity. In this study, we explored the relationship between platelet count, clinical characteristics, and the prognosis of patients with AAV. We divided 187 patients into two groups based on their platelet count. Clinicopathological data and prognostic information were retrospectively gathered from medical records. Univariate and multivariate regression analyses were used to identify risk factors for prognosis, including end-stage renal disease (ESRD) and mortality. The cutoff point for platelet count was set at 264.5 × 10^9^/L, as determined by the receiver operating characteristic (ROC) curve for predicting progression to ESRD in patients with AAV. We observed patients with low platelet count (platelets < 264.5 × 10^9^/L) had lower leukocytes, hemoglobin, complement, acute reactants, and worse renal function (*P* for eGFR < 0.001). They were also more likely to progress to ESRD or death compared to the high platelet count group (platelets > 264.5 × 10^9^/L) (*P* < 0.0001, *P* = 0.0338, respectively). Low platelet count was potentially an independent predictor of poor renal prognosis in the multivariate regression analysis [HR 1.670 (95% CI 1.019–2.515), *P* = 0.014]. Lower platelet count at diagnosis is associated with more severe clinical characteristics and impaired renal function. Therefore, platelet count may be an accessible prognostic indicator for renal outcomes in patients with AAV.

**Supplementary Information:**

The online version contains supplementary material available at 10.1007/s10238-024-01333-z.

## Introduction

Antineutrophil cytoplasmic antibody (ANCA)-associated vasculitis (AAV) is a group of complex autoimmune diseases characterized by the vasculitis, endothelial injury, and tissue damage as well as the presence of serum autoantibodies targeting myeloperoxidase (MPO-ANCA) or proteinase 3 (PR3-ANCA) [1, 2].

Platelets, one of the many components of peripheral blood, are primarily produced by mature megakaryocytes in the bone marrow with a diameter of 2–3 μm [3–6]. They have a lifespan of 7–14 days and undergo continuous apoptosis and regeneration to maintain a normal blood concentration of (100–300) × 10^9^/L. While their critical role in hemostasis and thrombosis is well-established, recent evidence has also identified platelets as key players in immunity, inflammation, neoangiogenesis, and tumor metastasis [4, 7, 8].

Platelet deposition in the glomerular and vascular structures of kidney tissue has been observed in various renal diseases [9]. Recently, increasing research has demonstrated that platelets may participate in the progression of autoimmune diseases including AAV [10]. Exactly, interactions between platelets and megakaryocytes, mediated by multiple bioactive substances from both cells, can influence platelet counts in AAV [11]. Mechanistically, the crosstalk of activated platelets with neutrophil, the coagulation and complement system as well as the involvement of platelet-derived inflammatory microparticles contributed to the progression of AAV [12–17]. Besides, a retrospective monocentric study performed by Park et al. indicated that a higher platelet-to-lymphocyte ratio at diagnosis was correlated with disease activity in AAV [18]. Previous studies have indirectly demonstrated that low platelet count may portend the poor prognosis in AAV, with patients experiencing worse outcomes—such as hypocomplementemia, treatment resistance, and glomerular immune complex deposition—typically presenting with lower platelet counts [19–22]. Apart from that, Sánchez et al. identified low platelet count along with low eGFR, advanced age, and male sex as predictors of death in their multivariate Cox model [23]. Conversely, Lee et al. proposed an index called the pan-immune-inflammation value (PIIV) calculated by platelet, neutrophil, monocyte, and lymphocyte counts. They found that a PIIV ≥ 1011.3 was an independent risk factor for all-cause mortality in AAV patients, which indirectly indicated that higher platelet count was associated with lower survival rate [24]. In addition, some clinical researches showed high platelet count may imply the activity of AAV, without directly linking to prognosis [11, 25].

Clearly, there is ongoing debate regarding the influence of platelet counts on the renal prognosis in AAV. The direct comparison of clinicopathological characteristics as well as outcome between patients with low and high platelet counts in AAV is still not well-defined. With this backdrop, our study aims to investigate whether platelet count was associated with the prognosis in AAV.

## Materials and methods

### Study population

A total of 187 patients with ANCA-associated vasculitis (AAV) were retrospectively recruited in the cohort from April 2016 to August 2021. The patients were sourced from the Department of Nephrology and Department of Rheumatology and Immunology of Xiangya Hospital Central South University and they were regularly followed up with a minimum of 15 months. All included patients were initially diagnosed without prior treatment with corticosteroids, immunosuppressive agents, or plasmapheresis based on the clinical and biological criteria of vasculitis according to the 2022 American College of Rheumatology (ACR) or the 2012 revised international Chapel Hill criteria [26, 27]. Patients were excluded if they were: (1) with medical conditions such as malignancies, hematological diseases, and inflammatory conditions other than AAV, (2) diagnosed with secondary vasculitis, (3) co-existing with other kidney diseases [18]. The study protocol was approved by the Medical Ethics Committee of Xiangya Hospital.

### Clinical and pathological data collection

Demographic, clinical, and pathological data at diagnosis were retrospectively collected from medical records. More exactly, the following information was gathered: (1) patients’ age and sex as demographic data; (2) assessment of organ involvement; (3) laboratory data including the type of ANCA, leukocyte counts, erythrocyte counts, hemoglobin, platelet counts, neutrophil counts, lymphocyte counts, monocyte counts, albumin, serum complement levels(C3 and C4), erythrocyte sedimentation rate (ESR), C-reactive protein (CRP), urine erythrocyte counts, proteinuria, urinary albumin-to-creatinine ratio (UACR), serum creatinine, blood urea nitrogen and estimated glomerular filtration rate (eGFR); 4) pathological data such as glomerular involvement condition, destruction of Bowman’s capsule, rupture of glomerular basement membrane (GBM), fibrinoid necrosis, granulomatous lesions, tubular atrophy, interstitial fibrosis and interstitial inflammatory cell infiltration. The serum ANCA level was measured by indirect immunofluorescence assay and antigen-specific ELISA for PR3-ANCA and MPO-ANCA [28]. The activity of AAV was assessed by the Birmingham Vasculitis Activity Score (BVAS) version 3 (scores from 0 to 63) [29]. The eGFR was calculated according to the Chronic Kidney Disease Epidemiology Collaboration (CKD-EPI) equation [30]. Among the whole cohort, 74 patients underwent renal biopsies and the Berden classification was used to evaluate the pathological lesions observed in the biopsy specimens [31]. In addition, all renal biopsy samples were independently assessed and scored by at least two pathologists who were blinded to the patient’s information. Glomerular immunofluorescence for the immune globulin and complement was measured and scored according to the extent of immunostaining, categorized as negative (–), trace( ±), mild (1 +), moderate (2 +), and strong (3 +). The latter two were regarded as positive for immune complex deposition [22]. We assessed tubulointerstitial involvement and interstitial infiltration according to the semi-quantitative scoring system: normal (scored 0), mild (scored 1) for < 25% impairment, moderate (scored 2) for 25–50% impairment, and severe (scored 3) for > 50% impairment [32].

### Definitions

The entire cohort was divided into two groups according to the platelet count cutoff point of 264.5 × 10^9^/L, which was defined through the ROC curve to predict progression to ESRD in patients with AAV. The outcome events included ESRD and death. ESRD was defined as eGFR < 15 ml/min per 1.73 m^2^ or the requirement for renal replacement therapy for more than 3 months, including hemodialysis, peritoneal dialysis, or renal transplantation[33].

### Statistical analysis

We conducted statistical analysis using SPSS software (version 26.0) and the figures were produced by GraphPad Prism software (version 8.0). Since all quantitative variables exhibited abnormal distribution, they were expressed as the median and interquartile range (IQR) and analyzed using the Mann–Whitney U test for comparing two samples while the Kruskal–Wallis H test followed by Tukey's test for multiple comparisons of multi-sample data. Box plots were used to visually represent continuous data, with vertical bars indicating the minimum and maximum values. Both categorical data and ranked data were represented as numbers and percentages, with the former compared using the chi-square test or Fisher’s exact tests, and the latter analyzed through the Mann–Whitney U test. We examined the correlation between two continuous variables by the Spearman correlation coefficient. Cumulative renal and overall survival curves were derived from the Kaplan–Meier method, and the differences between curves were measured using the log-rank test. Both univariable and multivariable Cox proportional hazards models were utilized to determine the link between low platelet count and renal outcome events. A two-sided *P* < 0.05 was regarded as statistically significant.

## Results

### Baseline clinicopathological characteristics and outcome of the cohort

All 187 patients enrolled from Xiangya Hospital Central South University were followed up until February 2023, with a median follow-up duration of 26 months (IQR 17-45). The baseline clinicopathological characteristics and outcomes of the cohort at diagnosis are described in Tables 1, 2, and 3.

**Table 1:** Clinical characteristics at AAV diagnosis according to platelet count

Characteristics	Entire cohort (*N* = 187)	Platelets < 264.5 × 10^9^/L [*n* = 111 (59%)]	Platelets > 264.5 × 10^9^/L [*n* = 76 (41%)]	*P*-value
Age (years), median (IQR)	64 (56–71)	63 (55–71)	65 (56–71)	0.471
Sex (male), *n* (%)	96 (51.3)	54 (48.6)	42 (55.3)	0.374
MPO-ANCA (or P-ANCA)	178 (95.2)	106 (95.5)	72 (94.7)	> 0.9
PR3-ANCA (or C-ANCA)	8 (4.3)	4 (3.6)	4 (5.3)	0.717
BVAS, median (IQR)	18 (14–20)	18 (14–20)	18 (14.25–20)	0.728
Organ involvement at diagnosis				
Systemic, *n* (%)	102 (54.5)	50 (45)	52 (68.4)	**0.002**
Cutaneous, *n* (%)	8 (4.3)	3 (2.7)	5 (6.6)	0.274
Mucous membranes/eyes, *n* (%)	7 (3.7)	4 (3.6)	3 (3.9)	> 0.9
ENT, *n* (%)	9 (4.8)	4 (3.6)	5 (6.6)	0.489
Lung, *n* (%)	172 (92)	102 (91.9)	70 (92.1)	> 0.9
Cardiovascular, *n* (%)	29 (15.5)	17 (15.3)	12 (15.8)	> 0.9
Digestive, *n* (%)	8 (4.3)	6 (5.4)	2 (2.6)	0.476
Renal, *n* (%)	183 (97.9)	111 (100)	72 (94.7)	**0.026**
Neurological, *n* (%)	21 (11.2)	8 (7.2)	13 (17.1)	**0.035**
Leukocytes (× 10^9^/L)	8 (6–10.8)	6.8 (5.5–8.4)	9.9 (8.05–12.83)	**< 0.001**
Erythrocytes (× 10^12^/L)	2.78 (2.38–3.31)	2.63 (2.28–3.08)	3.08 (2.63–3.47)	**< 0.001**
Hemoglobin (g/L)	83 (69–93)	79 (67–88)	86.5 (75–101)	**0.003**
Neutrophils (× 10^9^/L)	5.9 (4.3–8.6)	5.1 (3.8–6.8)	7.6 (5.68–10.1)	**< 0.001**
Lymphocytes (× 10^9^/L)	1.1 (0.8–1.5)	1 (0.7–1.3)	1.2 (0.8–1.8)	**0.025**
Monocytes (× 10^9^/L)	0.5 (0.4–0.8)	0.4 (0.3–0.6)	0.7 (0.5–0.9)	**< 0.001**
Albumin (g/L)	32.45 (28.8–36.6)	33.25 (29.73–36.9)	30.75 (27.28–34.53)	**0.025**
sC3 (mg/L)	783 (645–927.5)	730 (619–827)	888.5 (719.5–1085)	**< 0.001**
sC4 (mg/L)	243 (190.3–302)	228 (179–284)	267 (206.5–322.5)	**0.015**
ESR (mm/h)	74 (42.25–120)	59 (35–92)	104 (60.75–120)	**< 0.001**
CRP (mg/L)	21.4 (6.31–78.8)	13.1 (4.09–63.6)	38.6 (16.33–99.73)	**< 0.001**
Urine erythrocyte counts (/μl)	140 (30.1–344.75)	164.5 (56–445.67)	51.5 (15–241.56)	**0.001**
Proteinuria (g/24H)	1.41 (0.78–2.53)	1.62 (0.93–2.74)	1.08 (0.65–2)	**0.015**
UACR (g/g)	2.5 (1.45–5.84)	2.75 (1.68–6.62)	1.96 (1.22–5.74)	0.112
Serum creatinine (μmol/L)	381 (206.7–577)	489 (305.1–627)	247 (130.5–410)	**< 0.001**
Blood urea nitrogen (mmol/L)	17.6 (11.13–23.8)	19.5 (15.13–26.95)	13.2 (8.43–20.44)	**< 0.001**
eGFR (mL/min/1.73m^2^)	12.34 (6.81–24.91)	9.27 (5.9–15.98)	22.44 (10.67–42.84)	**< 0.001**

Values in bold correspond to significant values (*P* < 0.05)

**Table 2:** Pathological presentation at AAV diagnosis according to platelet count

Characteristics	Entire cohort (*N* = 187)	Platelets < 264.5 × 10^9^/L [*n* = 111 (59%)]	Platelets > 264.5 × 10^9^/L [*n* = 76 (41%)]	*P*-value
Underwent renal biopsy	74 (39.6)	42 (37.8)	32 (42.1)	0.558
Berden classification, *n* (%)				0.873
1 (focal)	15 (21)	7 (17)	8 (25)	–
2 (crescentic)	9 (12)	5 (12)	4 (13)	–
3 (mixed)	41 (56)	24 (59)	17 (53)	–
4 (sclerotic)	8 (11)	5 (12)	3 (9)	–
Glomerular involvement, median (IQR)				
% of crescentic glomeruli	46 (25–61)	48 (26–67.5)	45 (20–57.5)	0.526
% of sclerotic glomeruli	22 (12–41)	22 (15–43)	22 (8–41)	0.209
Destruction of Bowman’s capsule, *n* (%)	68 (93)	39 (95)	29 (91)	0.648
Rupture of GBM, *n* (%)	25 (34)	14 (34)	11 (34)	> 0.9
Fibrinoid necrosis, *n* (%)	33 (45)	18 (44)	15 (47)	0.8
Granulomatous lesions, *n* (%)	9 (12)	5 (12)	4 (13)	> 0.9
IC deposition, *n* (%)	24 (32.4)	19 (37.3)	5 (21.7)	0.187
IgA, *n* (%)	2 (2.7)	2 (3.9)	0 (0)	> 0.9
IgG, *n* (%)	4 (5.4)	3 (5.9)	1 (4.3)	> 0.9
IgM, *n* (%)	15 (20.3)	12 (23.5)	3 (13)	0.365
C3, *n* (%)	4 (5.4)	4 (7.8)	0 (0)	0.303
C1q, *n* (%)	0 (0)	0 (0)	0 (0)	-
C4, *n* (%)	2 (2.7)	2 (3.9)	0 (0)	> 0.9
*κ*, *n* (%)	2 (2.7)	2 (3.9)	0 (0)	> 0.9
*λ*, *n* (%)	5 (6.8)	3 (5.9)	2 (8.7)	0.643
Tubular atrophy integral, *n* (%)				0.19
0 (normal)	11 (15)	4 (10)	7 (22)	–
1 (mild)	38 (51)	25 (59)	13 (41)	–
2 (moderate)	25 (34)	13 (31)	12 (37)	–
3 (severe)	0 (0)	0 (0)	0 (0)	–
Interstitial fibrosis integral, *n* (%)				0.358
0 (normal)	17 (23)	12 (29)	5 (16)	–
1 (mild)	32 (43)	18 (42)	14 (44)	–
2 (moderate)	24 (33)	12 (29)	12 (37)	–
3 (severe)	1 (1)	0 (0)	1 (3)	–
Interstitial inflammatory cell infiltration integral, *n* (%)				**0.003**
0 (normal)	0 (0)	0 (0)	0 (0)	–
1 (mild)	33 (45)	25 (59)	8 (25)	–
2 (moderate)	31 (42)	15 (36)	16 (50)	–
3 (severe)	10 (13)	2 (5)	8 (25)	–

Values in bold correspond to significant values (*P* < 0.05)

**Table 3:** Main outcomes of AAV patients according to platelet count

Characteristics	Entire cohort (*N* = 187)	Platelets < 264.5 × 10^9^/L [*n* = 111 (59%)]	Platelets > 264.5 × 10^9^/L [*n* = 76 (41%)]	*P*-value
Follow-up duration (months), median (IQR)	26 (17–45)	24 (16–34)	34 (19–53)	**0.005**
KRT, *n* (%)				**0.001**
Never	96 (51.3)	45 (40.5)	51 (67.1)	–
< 3 months	20 (10.7)	12 (10.8)	8 (10.5)	–
> 3 months	71 (38)	54 (48.6)	17 (22.4)	–
KRT at least once, *n* (%)	91 (48.7)	66 (59.5)	25 (32.9)	**< 0.001**
ESRD, *n* (%)	133 (71.1)	95 (85.6)	38 (50)	**< 0.001**
1-year overall survival rates, *n* (%)	157 (84.0)	87 (78.4)	70 (92.1)	**0.012**
3-year overall survival rates, *n* (%)	130 (69.5)	70 (63.1)	60 (78.9)	**0.020**
Death, *n* (%)	65 (34.8)	45 (40.5)	20 (26.3)	**0.045**

Values in bold correspond to significant values (*P* < 0.05)

The median age of the whole participants was 64 years old (IQR 56-71) and 96 (51.3%) patients were male. Among the cohort, 178 (95.2%) patients were positive for myeloperoxidase-ANCA and 8 (4.3%) patients were positive for proteinase 3-ANCA. The median BVAS was 18 (IQR 14-20) at diagnosis in AAV. Kidney was the most commonly affected organ followed by lung and systemic involvement [172 (92%) and 102 (54.5%), respectively]. At the baseline, the median serum creatinine and eGFR were 381 μmol/L (IQR 206.7-577) and 12.34 ml/min per 1.73 m^2^ (IQR 6.81-24.91), with a median proteinuria of 1.41 g/24 h (IQR 0.78-2.53). Kidney biopsies were performed in 74 (39.6%) patients. According to the Berden classification to evaluate the pathological lesions in biopsy specimens, Class I (focal), Class II (crescentic), Class III (mixed), and Class IV (sclerotic) were found in 15 (21%), 9 (12%), 41 (56%), and 8 (11%) patients, respectively [31]. During follow-up, 133 (71.1%) and 65 (34.8%) patients progressed to ESRD and death, respectively, defined as mentioned above.

### Comparison of variables between patients with low and high platelet count

**Fig. 1:**
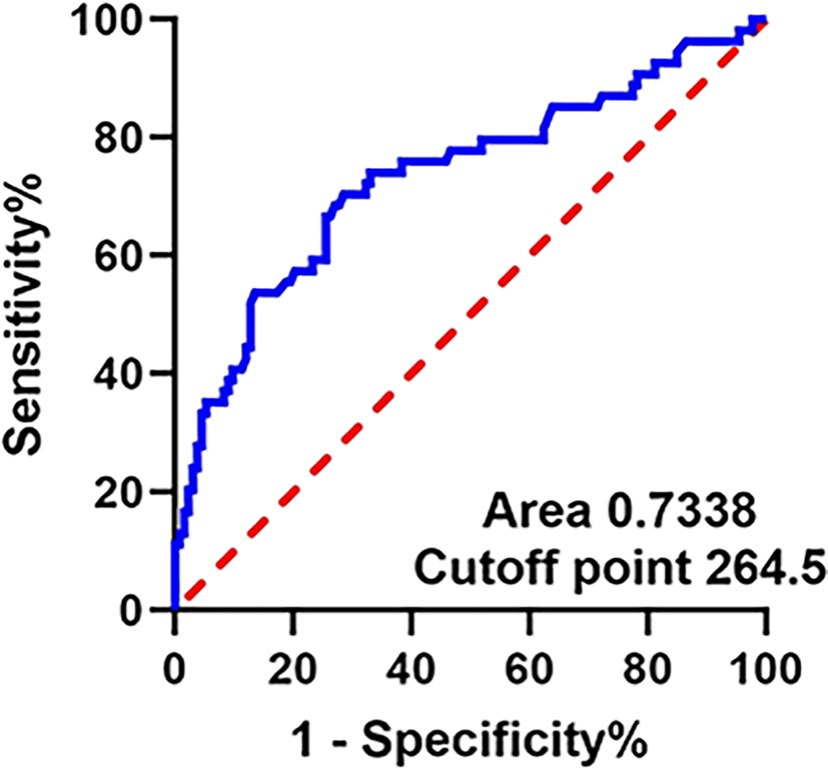
Receiver operating characteristic curve showing the sensitivity and specificity of platelet count for predicting progression to ESRD of AAV patients

We performed the ROC curve analysis with an area under the ROC curve (AUC) of 0.7338 and obtained the optimal platelet count cutoff of 264.5 × 10^9^/L for predicting progression to ESRD (Fig. 1). Among the 187 AAV patients, 111 (59%) patients presented with a low platelet count, and 76 (41%) patients had a high platelet count, in which forty-nine patients had a platelet count above the upper limitation of normal range 300 × 10^9^/L. Detailed contrast of characteristics between the two group of AAV patients at onset is shown in Tables 1, 2, and 3.

It was not statistically significant in demographic data with a median age of 63 years old (IQR 55-71) versus 65 years old (IQR 56-71) between patients with low and high platelet counts. Patients with low platelet count exhibited lower incidences of systemic (45% versus 68.4%; *P* = 0.002) and neurological (7.2% versus 17.1%; *P* = 0.035) manifestations while higher frequency of renal involvement (100% versus 94.7%; *P* = 0.026). Regarding laboratory results, AAV patients with lower platelet count presented with lower leukocytes [6.8 × 10^9^/L (IQR 5.5–8.4) versus 9.9 × 10^9^/L (IQR 8.05–12.83); *P* < 0.001], hemoglobin [79 g/L (IQR 67–88) versus 86.5 g/L (IQR 75–101); *P* = 0.003], serum complement levels [sC3 of 730 mg/L (IQR 619–827) versus 888.5 mg/L (IQR 719.5–1085); *P* < 0.001; sC4 of 228 mg/L (IQR 179–284) versus 267 mg/L (IQR 206.5–322.5); *P* = 0.015, respectively], ESR [59 mm/h (IQR 35–92) versus 104 mm/h (IQR 60.75–120); *P* < 0.001], CRP [13.1 mg/L (IQR 4.09–63.6) versus 38.6 mg/L (IQR 16.33–99.73); *P* < 0.001] as well as higher albumin [33.25 g/L (IQR 29.73–36.9) versus 30.75 g/L (IQR 27.28–34.53); *P* = 0.025]. In addition, compared to the cases with high platelet count, patients with low platelet count had lower eGFR [9.27 mL/min/1.73 m^2^ (IQR 5.9–15.98) versus 22.44 mL/min/1.73 m^2^ (IQR 10.67–42.84); *P* < 0.001] while more urine erythrocyte counts [164.5/μl (IQR 56–445.67) versus 51.5/μl (IQR 15–241.56); *P* = 0.001], proteinuria [1.62 g/24H (IQR 0.93–2.74) versus 1.08 g/24H (IQR 0.65–2.00); *P* = 0.015] and higher serum creatinine [489 μmol/L (IQR 305.1–627) versus 247 μmol/L (IQR 130.5–410); *P* < 0.001].

Regrettably, in terms of patients’ pathological findings, when examining the patients' pathological findings, no significant differences were identified in glomerular involvement, tubulointerstitial lesions, or the distribution of pathological classification between the patients with low and high platelet count—except for the interstitial inflammatory cell infiltration integral (*P* = 0.003). In regard to the Berden Classification, patients predominantly exhibited mixed ANCA-associated glomerulonephritis with a median platelet count of 252 × 10^9^/L (IQR 203–313). Similarly, there was still no statistical significance about platelet count at diagnosis in the four distinct pathological forms of AAV patients according to Berden Classification (Supplementary data, Fig. 1).

**Fig. 2:**
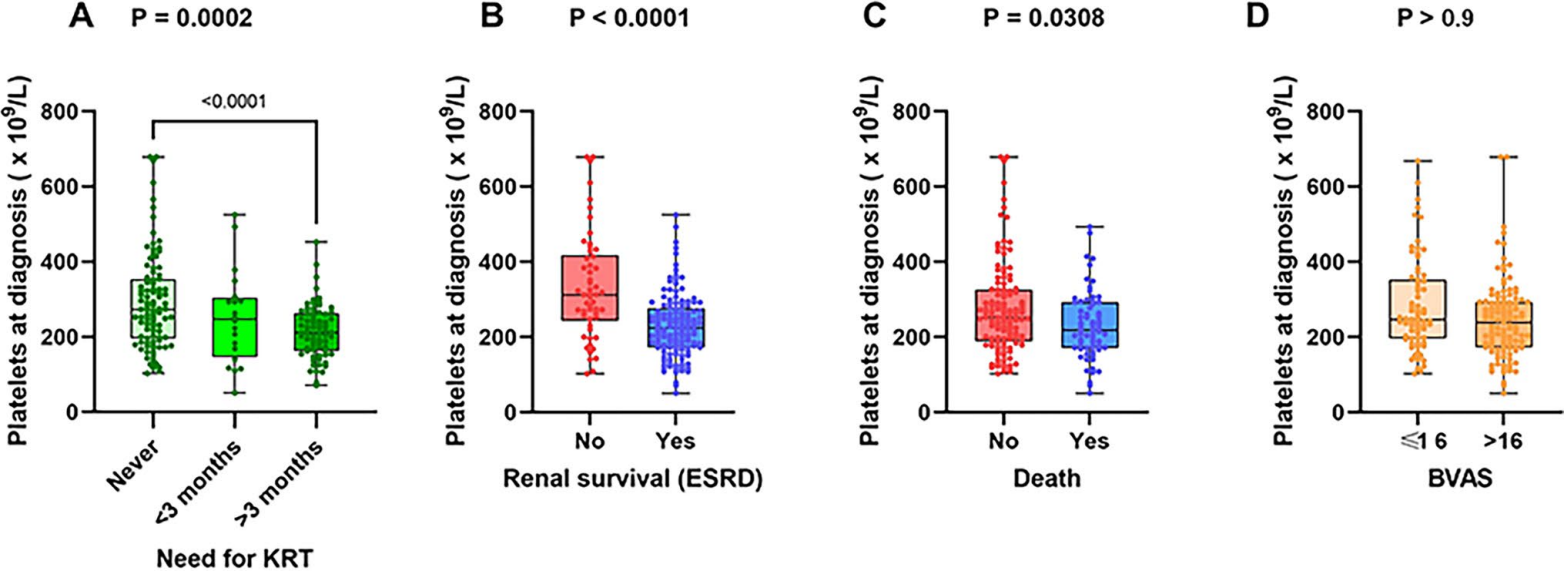
Platelet count according to relevant outcomes **A** Need for KRT during follow-up **B** Renal survival (ESRD) **C** death. **D** BVAS

Compared to those with high platelet count, patients with low platelet count were more prone to undergo kidney replacement therapy and had higher frequency of progression to ESRD (85.6% versus 50%; *P* < 0.001) accompanying with lower one-year and three-year overall survival rates (78.4% versus 92.1%; *P* = 0.012 and 63.1% versus 78.9%; *P* = 0.020, respectively). Consistently, patients with lower platelet counts exhibited comparatively higher percentage of death numbers than those with higher platelet counts (40.5% versus 26.3%; *P* = 0.045). In addition, we compared the circulating platelet counts at the onset in patients with varying disease activity levels and outcome statuses. Exactly, platelet levels were significantly lower in patients who needed KRT for more than 3 months and who progressed to ESRD as well as death (Fig. 2A, *P* < 0.0001; Fig. 2B, *P* < 0.0001; Fig. 2C, *P* = 0.0308; respectively). There was no significance of platelet counts in patients between BVAS ≤ 16 and BVAS > 16 (Fig. 2D, *P* > 0.9).

### Correlations of platelet count with clinical characteristics in AAV

We analyzed correlations between the count of platelet and clinical features at diagnosis in AAV. Platelet level showed no relevance with baseline parameters including age and BVAS. Obviously, platelet count was positively associated with leukocytes, serum complement level, inflammatory indicators (ESR and CRP), and eGFR (*r* = 0.4665, *P* < 0.0001; *r* for sC3 = 0.4564, *P* < 0.0001; *r* for sC4 = 0.2147, *P* = 0.0047; *r* for ESR = 0.4122, *P* < 0.0001; *r* for CRP = 0.2715, *P* = 0.0002; *r* = 0.3807, *P* < 0.0001; respectively), while negatively correlated with albumin and renal involvement parameters such as urine erythrocyte counts, proteinuria, UACR, serum creatinine, blood urea nitrogen (*r* = − 0.1774, *P* = 0.0163; *r* = − 0.2547, *P* = 0.0009; *r* = − 0.2102, *P* = 0.0076; *r* = − 0.1866, *P* = 0.0251; *r* = − 0.3814, *P* < 0.0001; *r* = − 0.4012, *P* < 0.0001; respectively). Collectively, our data suggested that the lower platelet level of AAV patients may be closely related to severer renal damage and worse renal outcomes (Table 4).

**Table 4:** Correlations of platelet count with laboratory findings in AAV patients

Characteristics	*r*-value	*P* value	Sig
Age (years)	− 0.0031	> 0.9	ns
BVAS	− 0.1192	0.1042	ns
Leukocytes (× 10^9^/L)	0.4665	** < 0.0001**	****
Erythrocyte s(× 10^12^/L)	0.3360	** < 0.0001**	****
Hemoglobin (g/L)	0.2378	**0.0011**	**
Neutrophils (× 10^9^/L)	0.3932	**< 0.0001**	****
Lymphocytes (× 10^9^/L)	0.3088	** < 0.0001**	****
Monocytes (× 10^9^/L)	0.4193	** < 0.0001**	****
Albumin (g/L)	− 0.1774	**0.0163**	*
sC3 (mg/L)	0.4564	**< 0.0001**	****
sC4 (mg/L)	0.2147	**0.0047**	**
ESR (mm/h)	0.4122	** < 0.0001**	****
CRP (mg/L)	0.2715	**0.0002**	***
Urine erythrocyte counts (/μl)	− 0.2547	**0.0009**	***
Proteinuria (g/24H)	− 0.2102	**0.0076**	**
UACR (g/g)	− 0.1866	**0.0251**	*
Serum creatinine (μmol/L)	− 0.3814	**< 0.0001**	****
Blood urea nitrogen (mmol/L)	− 0.4012	**< 0.0001**	****
eGFR (mL/min/1.73 m^2^)	0.3807	**< 0.0001**	****

Values in bold correspond to significant values (*P* < 0.05)

### AAV patients with low platelet count had poorer disease phenotypes and prognosis

**Fig. 3:**
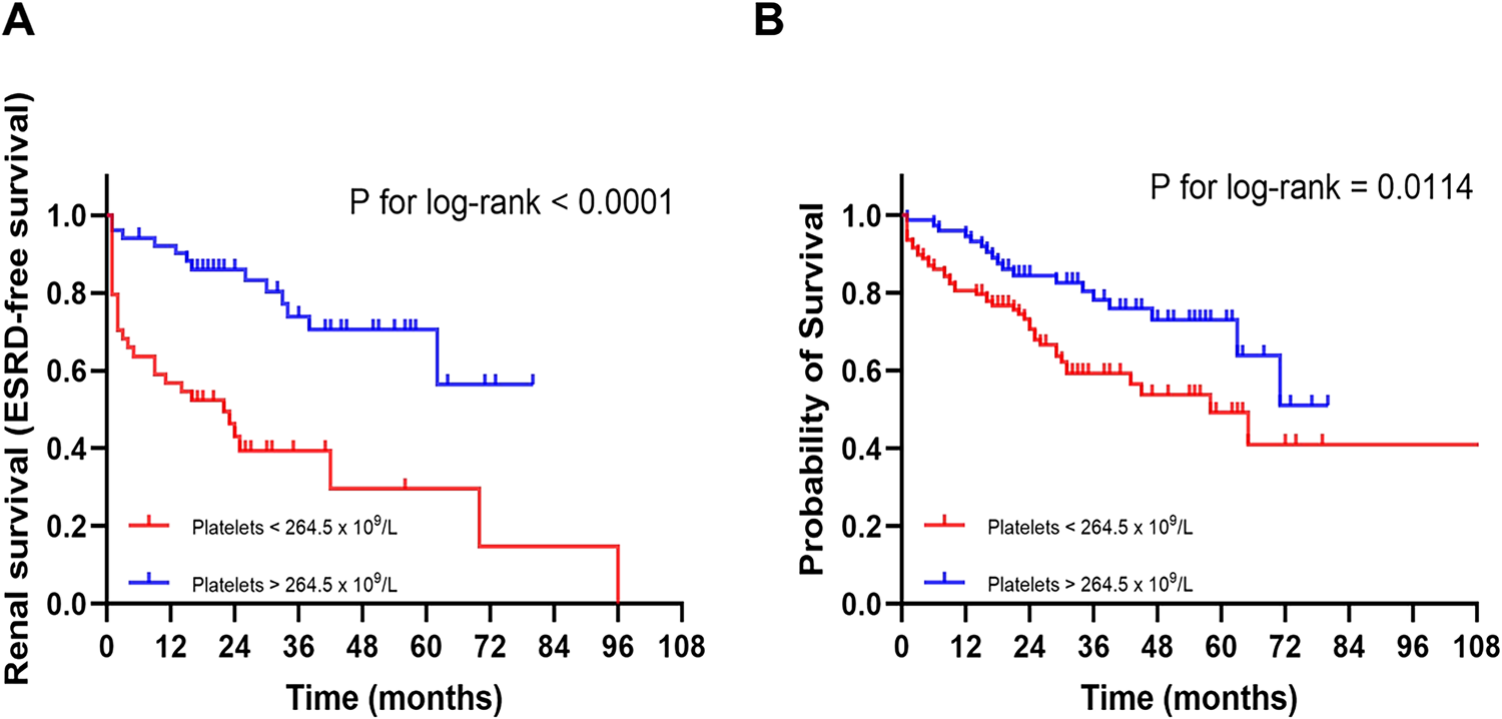
Kaplan–Meier curve for renal survival and all-cause mortality of AAV patients with low and high platelet count **A** renal survival and **B** overall survival according to platelet count. In red, patients with low platelet count (< 264.5 × 10^9^/L); in blue, patients with high platelet count (> 264.5 × 10.^9^/L)

Aiming to further verify the prognostic value of platelet count in patients with AAV, we next performed the Kaplan–Meier survival analysis. The results demonstrated that patients with low platelet counts had a worse overall survival rate and ESRD-free survival rate (Fig. 3A–B; *P* < 0.0001 and *P* = 0.0114, respectively).

**Fig. 4:**
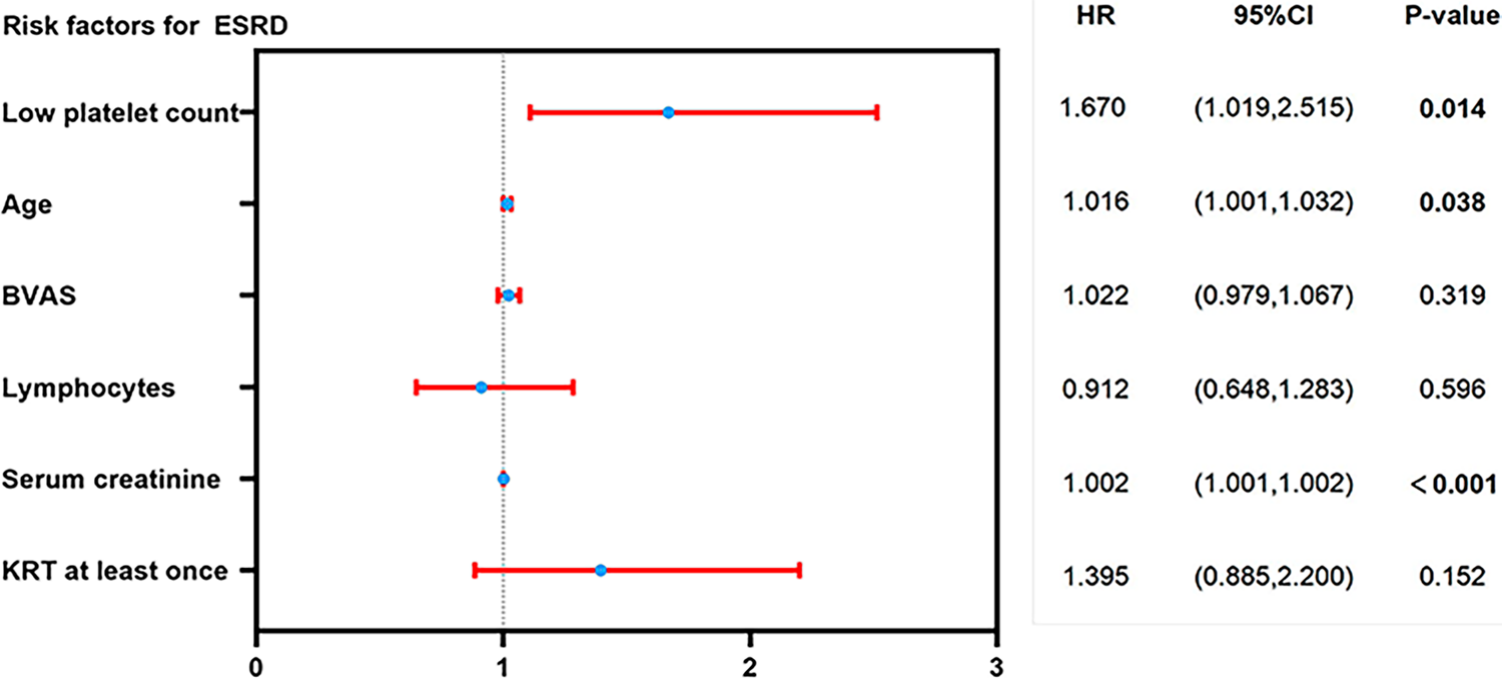
Forest plot of multivariate Cox model for ESRD prediction

In the univariate Cox regression analysis of predictors for ESRD in AAV, age, BVAS, erythrocytes, hemoglobin, low platelet count, lymphocytes, serum C3, serum creatinine, eGFR, histopathological classification, percentage of crescentic glomeruli, moderate interstitial fibrosis integral and KRT condition were predictors of progression to ESRD (Supplementary data, Table 1). However, in the multivariate analysis, only low platelet count [HR 1.670 (95% CI 1.019–2.515), *P* = 0.014], serum creatinine [HR 1.002 (95% CI 1.001–1.002), *P* < 0.001] and age [HR 1.016 (95% CI 1.001–1.032), *P* = 0.038] remained as significant risk factors for ESRD occurrence (Fig. 4 and Supplementary data, Table 1). In other words, low platelet count was associated with a high risk of progressing to ESRD in AAV. Furthermore, although platelet status was a predictor for death in the univariate regression analysis, no correlation was found between platelet count and overall survival rate in the multivariate analysis (data not shown).

## Discussion

In this study, we analyzed the clinicopathological features and prognoses in AAV from a novel perspective according to platelet count. Our results focused on the association between platelet count and renal outcome. In detail, we demonstrated that patients with low platelet count were usually accompanied with less systemic and neurological involvement, more renal involvement, lower leukocytes, complement, acute reactants, hemoglobin, worse renal function, and poorer prognoses within our cohort of individuals newly diagnosed with AAV. In line with this, our multivariate regression analysis indicated that low platelet count may serve as an independent indicator of worse renal prognosis in AAV.

Previous studies have reported that most active AAV patients had platelet counts within or above the normal range, which is consistent with our study, namely, only three of all patients had a platelet count of lower than 100 × 10^9^/L [25, 34]. Willeke et al. [11] demonstrated that platelet count was related to the activity of disease in AAV. However, we did not observe the association between platelet count and BVAS score in our study. Fukui et al. reported that AAV patients with hypocomplementemia often presented with low platelet count [20]. And hypocomplementemia at diagnosis was a significant risk factor for poorer prognosis in AAV [20]. Similarly, a retrospective study from our center by Huang et al. showed that patients with treatment resistance usually had worse initial renal function levels and lower platelet counts [21]. A German single-center observational study suggested that lower platelet counts correlated closely with kidney injury, specifically with tubulointerstitial injury [34]. And this subset of AAV patients presented with lower overall survival and prolonged hospitalization [34]. Another European multiple-center research also proved that multiple variables at diagnosis including low platelet count were regarded as predictors of death in their multivariate Cox model [23]. Despite these insights, previous studies have not systematically compared clinical characteristics and outcomes between AAV patients with low and high platelet counts. Given this, we visually observed that patients with relatively lower platelet counts had worse clinical characteristics, renal function, and prognoses. And specifically, in our study, the lower frequency of systemic involvement may explain the lower ESR and CRP levels reflecting systemic condition, while the increased urine erythrocyte counts may result in decreased hemoglobin in patients with relatively lower platelet counts. Additionally, low platelet count along with serum creatinine and age were identified as potential risk factors for ESRD in multivariate regression analysis.

Unfortunately, as expected, no statistical difference was found in terms of renal histopathological findings, including glomerular lesions and tubulointerstitial involvement, except for the interstitial inflammatory cell infiltration integral, between the two groups of patients. In this regard, we speculate that one possible reason for this result was the limited number of cases in which patients agreed to undergo renal aspiration biopsy.

So what are the hidden and moderate mechanisms of the phenomenon that platelet counts of most active AAV patients were within or above the normal range? On the other hand, how can we explain the association between platelet count and prognosis of AAV patients? Combined with previous literature research, we put forward some thoughts as follows.

Platelets, crucial in hemostasis, are increasingly recognized for their important role in inflammation [8, 35, 36]. In the acute inflammatory phase of AAV, activated platelet can release microparticles rich in multiple proinflammatory cytokines, contributing to the development of the disease [17]. Baier et al. proposed that the reactive increase in platelets could lead to elevated platelet count in active AAV cases [34]. In our study, forty-nine patients had a platelet count above the upper limitation of the normal range 300 × 10^9^/L among the 187 AAV patients. Willeke et al. speculated that the interaction between platelet-derived sCD40L and MMP-9 may facilitate the increase in platelet production in active AAV patients [11]. Apart from that, established literature demonstrated that platelet-derived CCL5 increased megakaryocyte ploidy, subsequent proplatelet production, and final increase in platelet count in a CCR5-dependent manner through apoptosis suppression during acute inflammation [37]. Hence, the effect of platelet-derived CCL5-megakaryocyte-derived-CCR5 axis on megakaryocyte may also promote the increased platelet count in AAV but remains to be confirmed.

It has been previously described that lower platelet counts in patients with severe granulomatosis with polyangiitis (GPA) might be caused by local platelet aggregation or consumption due to mechanisms similar to disseminated intravascular coagulation (DIC) or capillaritis [11]. As is well-known, neutrophils are indispensable for the pathogenesis of AAV. Etulain et al. proposed that activated platelets can interact with neutrophils and further contribute to the formation of neutrophil extracellular traps (NETs) [38]. NETs, in turn, will promote platelet adhesion and aggregation through several aspects as follows. Firstly, the fibers of NETs could directly bind to platelets and boost the aggregation of platelets, while the histones of NETs can further stimulate platelet activation, creating a positive feedback [39, 40]. Additionally, NETs can activate the coagulation system, leading to thrombin production. Thrombin can not only boost the release of vWF from the endothelium to mediate platelet adhesion and aggregation but also further activate the alternative complement pathway and facilitate the production of C5a [10, 14]. It is reported that C5a can induce platelet adhesion and aggregation on microvascular endothelial cells in patients with atypical hemolytic uremic syndrome and COVID-19, suggesting a potential role in AAV that awaits further validation [41]. Moreover, the activated platelets can prompt the generation of monomeric CRP, which induces the release of mitochondrial DNA from platelets. The platelet-released mitochondrial DNA can next increase the activation and aggregation of platelets, cause the activation of the coagulation system, and augment the pathogenicity of ANCA in AAV [15, 16, 42]. Taken together, we hypothesize that activated platelets interact with leukocyte and endothelial cells, presenting with a relatively lower platelet level and accelerating the development of AAV through the mechanisms mentioned above, thereby leading to a worse prognosis.

To the best of our knowledge, this is the first research to explore the clinicopathological features and outcomes of AAV patients with low and high platelet counts. Similar to most clinical studies, there are also some limitations decided by the nature of retrospective, unicentric, small-sample capacity research. Therefore, it is urgently needed to further demonstrate in multi-center institutions in favor of our results.

## Conclusions

In summary, in our study, we found that the clinical severity in patients with low platelet count at onset is inferior to those with high platelet count and low platelet count may serve as an independent indicator of a worse prognosis in AAV.

## Supplementary Information

Below is the link to the electronic supplementary material.

Supplementary file1 (DOCX 121 kb)

## Data Availability

The datasets used and/or analyzed during the current study are available from the corresponding author on reasonable request.
