# Supplementary material for: Low platelet count at diagnosis of anti-neutrophil cytoplasmic antibody-associated vasculitis is correlated with the severity of disease and renal prognosis
Source: Clin Exp Med. 2024 Apr 5;24(1):70. doi: 10.1007/s10238-024-01333-z (PMC10997538; doi:10.1007/s10238-024-01333-z)
Supplement: Supplementary file 1 — Supplementary file1 (DOCX 121 kb) [file 10238_2024_1333_MOESM1_ESM.docx]

Low platelet count at diagnosis of anti-neutrophil cytoplasmic antibody-associated vasculitis is correlated with the severity of disease and renal prognosis

Yanli Jin^1^ · Fangyuan Wang^1^ · Jiale Tang^2^ · Liying Luo^2^ · Lingyu Huang^1^ · Fangyu Zhou^1^ · Enyu Qi^1^ · Xinyue Hu^2^ · Shuanglinzi Deng^2^ · Huan Ge^2^ · Yuanyuan Jiang^3^ · Juntao Feng^2^ · Xiaozhao Li^1*^

^1^Department of Nephrology, Xiangya Hospital, Central South University, Changsha, China

^2^Center of Respiratory Medicine, Xiangya Hospital, Central South University, Changsha, China

^3^Department of Laboratory Medicine, Xiangya Hospital, Central South University, Changsha, China

***Correspondence:**Xiaozhao Li

Tel: +8613507474554
E-mail address: [lixiaozhao@csu.edu.cn](mailto:lixiaozhao@csu.edu.cn)

Postal address: No.87 Xiangya Road, Kaifu District, Changsha, Hunan Province, China

**Journal name:** Clinical and Experimental Medicine

**Supplementary Material**


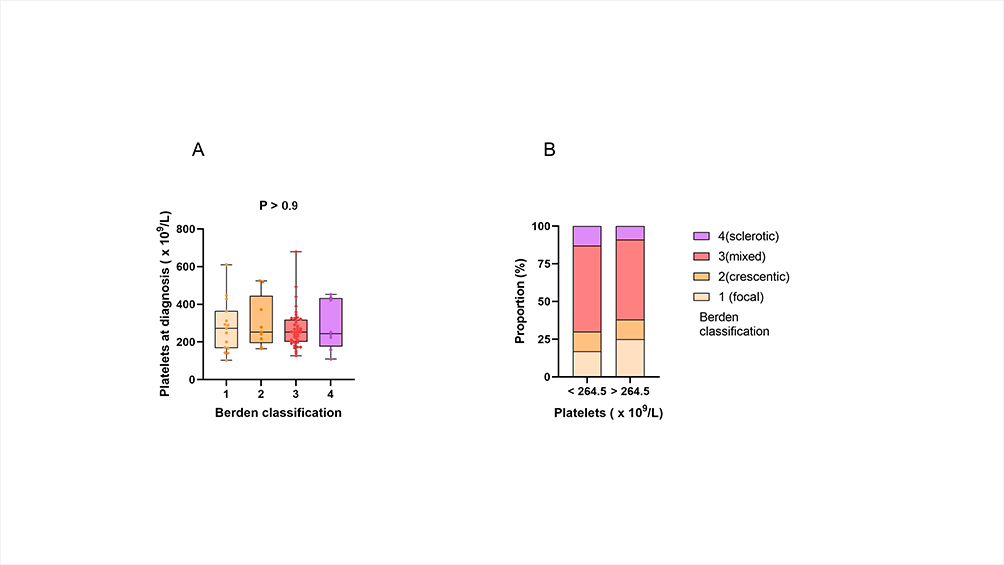


**Supplementary Fig. 1** Relation between platelet levels and pathological findings (A) Platelet counts according to Berden classification (B) Distribution of Berden classification according to thrombocytosis status

**Supplementary Table 1.** Cox regression analysis of predictors for renal outcome in patients with AAV

|  | **Time to ESRD**  N =133(71.1%) | | | | | | |
| --- | --- | --- | --- | --- | --- | --- | --- |
|  | **Univariate analysis** | | |  | **Multivariate analysis** | | |
| Characteristics | **HR** | **95% CI** | **p-value** |  | **HR** | **95% CI** | **p-value** |
| Demographic data at diagnosis |  |  |  |  |  |  |  |
| Age (years) | 1.017 | (1.003, 1.032) | 0.021 |  | 1.016 | (1.001, 1.032) | 0.038 |
| Sex (male) | 0.99 | (0.704, 1.394) | 0.956 |  |  |  |  |
| ANCA at diagnosis |  |  |  |  |  |  |  |
| MPO-ANCA (or P-ANCA) | 1.489 | (0.607, 3.653) | 0.385 |  |  |  |  |
| PR3-ANCA (or C-ANCA) | 0.558 | (0.205, 1.520) | 0.254 |  |  |  |  |
| Vasculitis activity at diagnosis |  |  |  |  |  |  |  |
| BVAS | 1.039 | (1.002, 1.076) | 0.038 |  | 1.022 | (0.979, 1.067) | 0.319 |
| Organ involvement at diagnosis |  |  |  |  |  |  |  |
| Systemic | 0.856 | (0.607, 1.205) | 0.373 |  |  |  |  |
| Cutaneous | 0.885 | (0.362, 2.166) | 0.789 |  |  |  |  |
| Mucous membranes/eyes | 1.103 | (0.451, 2.697) | 0.830 |  |  |  |  |
| ENT | 1.053 | (0.490, 2.259) | 0.895 |  |  |  |  |
| Lung | 1.645 | (0.767, 3.527) | 0.201 |  |  |  |  |
| Cardiovascular | 1.336 | (0.858, 2.081) | 0.200 |  |  |  |  |
| Digestive | 0.788 | (0.320, 1.938) | 0.604 |  |  |  |  |
| Renal | 21.631 | (0.268, 1743.76) | 0.170 |  |  |  |  |
| Neurological | 0.735 | (0.405, 1.331) | 0.309 |  |  |  |  |
| Biological presentation at diagnosis |  |  |  |  |  |  |  |
| [Leukocyte](http://dict.youdao.com/w/leukocyte/#keyfrom=E2Ctranslation)s ( x 10^9^/L) | 0.986 | (0.959, 1.015) | 0.345 |  |  |  |  |
| [Erythrocyte](http://dict.youdao.com/w/erythrocyte/#keyfrom=E2Ctranslation)s ( x 10^12^/L) | 0.568 | (0.433, 0.744) | < 0.001 |  |  |  |  |
| [Hemoglobin](http://dict.youdao.com/w/hemoglobin/#keyfrom=E2Ctranslation) (g/L) | 0.983 | (0.974, 0.993) | 0.001 |  |  |  |  |
| Platelets < 264.5 x 10^9^/L | 2.342 | (1.589, 3.451) | < 0.001 |  | 1.670 | (1.019, 2.515) | 0.014 |
| Neutrophils ( x 10^9^/L) | 1.003 | (0.966, 1.042) | 0.870 |  |  |  |  |
| Lymphocytes ( x 10^9^/L) | 0.677 | (0.493, 0.929) | 0.016 |  | 0.912 | (0.648, 1.283) | 0.596 |
| Monocytes ( x 10^9^/L) | 0.927 | (0.631, 1.360) | 0.697 |  |  |  |  |
| Albumin (g/L) | 0.989 | (0.961, 1.018) | 0.444 |  |  |  |  |
| sC3 (mg/L) | 0.998 | (0.998, 0.999) | < 0.001 |  |  |  |  |
| sC4 (mg/L) | 0.999 | (0.997, 1.001) | 0.150 |  |  |  |  |
| Acute reactants at diagnosis |  |  |  |  |  |  |  |
| ESR (mm/h) | 0.997 | (0.992, 1.001) | 0.172 |  |  |  |  |
| CRP (mg/L) | 1.001 | (0.997, 1.004) | 0.683 |  |  |  |  |
| [Urine erythrocyte](http://dict.youdao.com/w/urine%20erythrocyte/#keyfrom=E2Ctranslation) counts (/μl) | 1.000 | (1.000, 1.000) | 0.361 |  |  |  |  |
| Proteinuria (g/24H) | 1.062 | (0.960, 1.174) | 0.242 |  |  |  |  |
| UACR (g/g) | 1.009 | (0.996, 1.021) | 0.185 |  |  |  |  |
| Serum creatinine (μmol/L) | 1.002 | (1.001, 1.003) | < 0.001 |  | 1.002 | (1.001, 1.002) | < 0.001 |
| Blood urea nitrogen (mmol/L) | 1.003 | (1.000, 1.006) | 0.070 |  |  |  |  |
| eGFR (mL/min/1.73m^2^) | 0.919 | (0.897, 0.940) | < 0.001 |  |  |  |  |
| Pathological findings |  |  |  |  |  |  |  |
| Berden classification |  |  |  |  |  |  |  |
| 1 (focal) | - | - | **-** |  |  |  |  |
| 2 (crescentic) | 4.865 | (1.392, 16.996) | 0.013 |  |  |  |  |
| 3 (mixed) | 2.947 | (1.025, 8.470) | 0.045 |  |  |  |  |
| 4 (sclerotic) | 3.870 | (1.082, 13.837) | 0.037 |  |  |  |  |
| Glomerular involvement |  |  |  |  |  |  |  |
| % of crescentic glomeruli | 1.018 | (1.005, 1.031) | 0.006 |  |  |  |  |
| % of sclerotic glomeruli | 1.010 | (0.997, 1.023) | 0.142 |  |  |  |  |
| Destruction of Bowman’s capsule | 1.747 | (0.421, 7.239) | 0.442 |  |  |  |  |
| Rupture of GBM | 1.544 | (0.828, 2.878) | 0.172 |  |  |  |  |
| Fibrinoid necrosis | 1.291 | (0.707, 2.357) | 0.406 |  |  |  |  |
| Granulomatous lesions | 1.275 | (0.537, 3.024) | 0.582 |  |  |  |  |
| Tubular atrophy integral |  |  |  |  |  |  |  |
| 0 (normal) | - | - | - |  |  |  |  |
| 1 (mild) | 0.884 | (0.373, 2.097) | 0.780 |  |  |  |  |
| 2 (moderate) | 1.234 | (0.513, 2.971) | 0.639 |  |  |  |  |
| 3 (severe) | - | - | - |  |  |  |  |
| Interstitial fibrosis integral |  |  |  |  |  |  |  |
| 0 (normal) | - | - | - |  |  |  |  |
| 1 (mild) | 0.684 | (0.337, 1.389) | 0.284 |  |  |  |  |
| 2 (moderate) | 0.397 | (0.174, 0.905) | 0.028 |  |  |  |  |
| 3 (severe) | - | - | 0.976 |  |  |  |  |
| Interstitial inflammatory cell infiltration integral |  |  |  |  |  |  |  |
| 1 (mild) | - | - | - |  |  |  |  |
| 2 (moderate) | 0.615 | (0.312, 1.213) | 0.160 |  |  |  |  |
| 3 (severe) | 1.321 | (0.577, 3.026) | 0.510 |  |  |  |  |
| Outcomes |  |  |  |  |  |  |  |
| KRT |  |  |  |  |  |  |  |
| Never | - | - | **-** |  |  |  |  |
| <3months | 3.178 | (1.830, 5.519) | < 0.001 |  |  |  |  |
| >3months (ESRD) | 2.700 | (1.836, 3.969) | < 0.001 |  |  |  |  |
| KRT at least once | 2.784 | (1.921, 4.036) | < 0.001 |  | 1.395 | (0.885, 2.200) | 0.152 |

Values in bold correspond to significant values (P < 0.05)
